# Bibliographical Department

**Published:** 1881-11

**Authors:** 


### BIBLIOGRAPHICAL DEPARTMENT.

The Popular Science Monthly for November, one of our most valued exchanges, is, as usual, full of good things. Herbert Spencer continues his researches on the Development of Political Institutions, with a paper on "The Industrial Type of Society." Dr. Oswald has another of his interesting articles on Physical Education. Sir John Lubbock furnishes an address full of historical facts on a Half-Century of Science, and the remainder of the magazine is filled with papers on the Deterioration of American Oyster Beds, a subject in which all of us are interested; Volcanoes, The Duration of Human Life, Worry, and American Climate and Character. The portrait in this month's issue is of Prof. Geo. J. Brush, the new President of the American Association for the Advancement of Science.

Like all of the Appletons' publications, the *Monthly* is all that can be desired in paper, printing and illustrations, and we gladly add it to our exchange list.

Sixteenth Annual Address before the Massachusetts Dental Society, by Albion M. Dudley, D. D. S., pp. 21, Salem, Mass., 1881. The author compliments us with a copy.

*The Herald of Dentistry* is another new quarterly. Nos. 1 and 2 received. Thomas O. Oliver, M. D., editor, Chas. L. Shunber, publisher and proprietor, 96 Nassau Street, New York; price $1.00 per annum.

The College Record is the title of a new four-page weekly published at St. Joseph, Mo., by F. C. Hoyt, M. D., editor. It is published in the interests of the St. Joseph College of Physicians and Surgeons.

*The Physicians' Visiting List for* 1882. *Thirty-first year of its publication. Philadelphia: Lindsay & Blakiston.* Contains the metric system in comparison with our present system of apothecaries' weights and measures, and the doses in both. Its long continued annual publication is evidence that it is a pocket gem to the physician.

